# Intelligent Segmentation of Urban Building Roofs and Solar Energy Potential Estimation for Photovoltaic Applications

**DOI:** 10.3390/jimaging11100334

**Published:** 2025-09-25

**Authors:** Junsen Zeng, Minglong Yang, Xiujuan Tang, Xiaotong Guan, Tingting Ma

**Affiliations:** 1 Faculty of Land Resource Engineering, Kunming University of Science and Technology, Kunming 650093, China; zengjunsen@stu.kust.edu.cn (J.Z.);; 2 Engineering Research Center for Application of Spatial Information Mapping Technology in Plateau Mountainous Areas, Yunnan Provincial Universities, Kunming 650093, China; 3 Institute of Surveying and Mapping of Kunming City, Kunming 650091, China

**Keywords:** deep learning, semantic segmentation, distributed photovoltaic, building extraction, multi-model prediction result fusion, shadow occlusion, power generation calculation

## Abstract

To support dual-carbon objectives and enhance the accuracy of rooftop distributed photovoltaic (PV) planning, this study proposes a multidimensional coupled evaluation framework that integrates an improved rooftop segmentation network (CESW-TransUNet), a residual-fusion ensemble, and physics-based shading and performance simulations, thereby correcting the bias of conventional 2-D area–based methods. First, CESW-TransUNet, equipped with convolution-enhanced modules, achieves robust multi-scale rooftop extraction and reaches an IoU of 78.50% on the INRIA benchmark, representing a 2.27 percentage point improvement over TransUNet. Second, the proposed residual fusion strategy adaptively integrates multiple models, including DeepLabV3+ and PSPNet, further improving the IoU to 79.85%. Finally, by coupling Ecotect-based shadow analysis with PVsyst performance modeling, the framework systematically quantifies dynamic inter-building shading, rooftop equipment occupancy, and installation suitability. A case study demonstrates that the method reduces the systematic overestimation of annual generation by 27.7% compared with traditional 2-D assessments. The framework thereby offers a quantitative, end-to-end decision tool for urban rooftop PV planning, enabling more reliable evaluation of generation and carbon-mitigation potential.

## 1. Introduction

Against the backdrop of the global energy transition and China’s “carbon peaking and carbon neutrality goals” (the “dual carbon” strategy), the development of renewable energy has become a core strategy for addressing the energy crisis and climate change [1]. Photovoltaic (PV) power generation, with its advantages of low pollution, low energy consumption, and low cost, has emerged as a key focus of green energy development worldwide [2,3]. High-precision assessment and potential estimation of urban rooftop PV resources constitute a critical component of renewable energy planning [4]. China has set clear targets for carbon peaking by 2030 and carbon neutrality by 2060, identifying distributed photovoltaics as an essential pathway for energy transition [5]. Rooftop PV systems are prioritized in distributed PV deployment due to their high land-use efficiency and strong capacity for local consumption [6]. However, the large-scale deployment of rooftop PV remains constrained by insufficient accuracy in rooftop resource identification, limited methods for solar potential estimation, and a lack of scientific basis for spatial planning, hindering its further development [7]. In response, the National Energy Administration of China launched a county-level pilot program for distributed rooftop PV in 2021, emphasizing the importance of intelligent evaluation technologies in PV planning [8].

In recent years, accurate segmentation and effective area calculation of building rooftops have remained one of the key challenges in rooftop photovoltaic (PV) potential assessment [9]. Current research primarily evolves along two trajectories: single-model segmentation and multi-model ensemble methods. In the domain of single-model segmentation, numerous advanced algorithms have yielded promising results in specific application scenarios. For example, Wu et al. [10] proposed an improved U-Net-based approach for rooftop extraction, enhancing feature representation and edge accuracy through the incorporation of spatial attention mechanisms. Similarly, Jenila and Varalakshmi [11] introduced an optimized Mask R-CNN framework for building footprint detection in high-resolution aerial imagery, which improves the segmentation of irregular rooftops and complex structures through enhanced region proposal networks. Nevertheless, these models often exhibit inherent structural biases toward specific rooftop materials, slopes, and other attributes, which limits their generalization across diverse urban environments. Such shortcomings inevitably introduce segmentation inaccuracies that propagate into subsequent PV potential estimations, thereby compromising overall reliability.

To mitigate the constraints of single-model approaches, researchers have begun to explore multi-model ensemble strategies. Xiang et al. [12] and Yu [13] adopted majority voting methods for fusing predictions from multiple models, improving segmentation stability to some extent in specific scenarios such as mining and urban rooftop extraction. Nonetheless, the field remains relatively underexplored—particularly in designing intelligent fusion mechanisms that effectively harness the complementary strengths of heterogeneous model architectures. Although techniques such as pixel-level global majority voting can sometimes enhance segmentation accuracy, they frequently exhibit instability and inconsistent performance. Such methods treat all model predictions equally, failing to adapt to spatial variations in model performance or to suppress erroneous contributions from underperforming models. As a result, error accumulation—especially around building boundaries and in occluded regions—persists as a critical challenge, often diminishing the advantages of superior models and constraining the robustness of the fused output.

At the level of PV potential estimation, most existing studies oversimplify actual deployment conditions by depending predominantly on two-dimensional rooftop area calculations while neglecting essential three-dimensional physical factors such as dynamic inter-building shading and rooftop equipment obstruction. For instance, Qi et al. [14] developed a regional assessment model based on remote sensing and deep learning, focusing on two-dimensional roof plane extraction for large-scale PV potential estimation. Likewise, Xu et al. [15] proposed a photovoltaic resource assessment method through roof usable area extraction using image segmentation, yet their approach still primarily relies on two-dimensional surface analysis without fully incorporating spatial and shading constraints. Such simplifications lead to systematically inflated energy generation estimates, creating a substantial gap between theoretical assessments and real-world application needs. Consequently, there is a critical demand for more sophisticated methodologies that incorporate refined geometric modeling, dynamic shading analysis, and realistic spatial exclusion criteria.

To address the intertwined challenges of insufficient rooftop segmentation accuracy, limited generalization of single models, and the absence of physical constraints in urban PV resource assessment, this study establishes a three-stage progressive evaluation framework. First, an improved multi-scale feature fusion network is employed to achieve high-precision rooftop segmentation. Second, a residual fusion strategy based on a modified majority voting method is developed to integrate predictions from multiple models, overcoming the inherent limitations of single-model predictions. Finally, the framework incorporates three-dimensional physics-based simulations to achieve accurate assessment of rooftop PV resources. This end-to-end technical approach provides comprehensive support for city-level PV resource surveys and carbon emission reduction calculations, facilitating the precise deployment of distributed PV systems and the dynamic, coordinated management of building carbon peaking targets.

## 2. Methods

### 2.1. Technical Workflow for Rooftop PV Resource Potential Assessment

**Figure 1:**
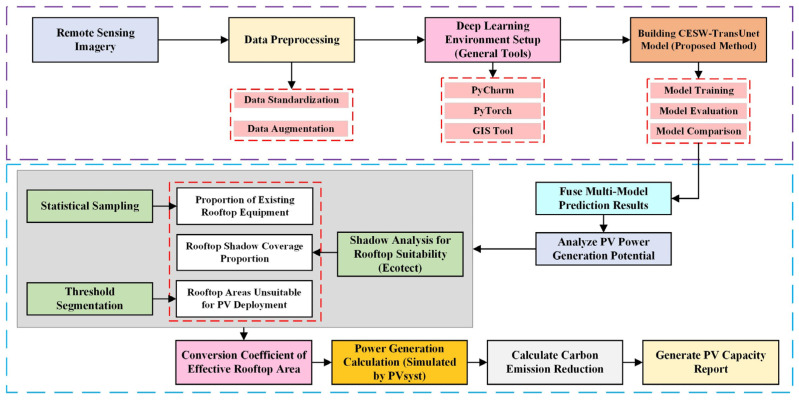
Workflow of the proposed multidimensional framework for urban rooftop PV potential assessment. The process begins with data preprocessing and model development, progresses to rooftop extraction and refinement by integrating physical constraints (equipment occupancy, dynamic shading, unsuitable areas), and concludes with energy simulation and carbon reduction calculation.

This study presents a framework for assessing rooftop photovoltaic (PV) potential by integrating deep learning with physical constraints. The workflow is shown in Figure 1. First, remote sensing images are preprocessed, and a CESW-TransUNet model is trained to extract rooftop contours, with multi-model fusion enhancing segmentation accuracy. Physical constraints are then applied to refine the area estimate, including statistical sampling of existing equipment occupancy, dynamic shading simulation, and exclusion of regions smaller than 2 m^2^. Finally, the effective rooftop area is used in PVsyst to simulate energy generation, and the resulting output is converted into carbon reduction potential based on regional grid emission factors, culminating in a comprehensive PV capacity and environmental impact assessment.

### 2.2. Construction of the Semantic Segmentation Network Model

To address the challenge of limited rooftop prediction accuracy in semantic segmentation models caused by the diversity of building types and the complexity of urban scenes in remote sensing imagery, we develop a novel Convolution-Enhanced Swin-Window based TransUNet (CESW-TransUNet) architecture that incorporates triple-structure optimization, building upon the original TransUNet framework [16]. This model synergistically leverages the advantages of convolutional neural networks and Transformer-based representations through a three-stage progressive architecture.

In the encoding stage, ResNet50 is utilized to extract multi-level local features, and the standard Vision Transformer (ViT) module is replaced by a Convolution-Enhanced Swin Block (CESW-Block) to strengthen convolutional representation. The intermediate stage integrates convolution-enhanced modules, including the CESW-Block, and implements Multi-Scale Channel Attention (MSCA) to enhance cross-scale semantic modeling. During the decoding stage, Global-guided Cross-level Feature Fusion (GCFF) and a feature reconstruction strategy are adopted to recover spatial details.

**Figure 2:**
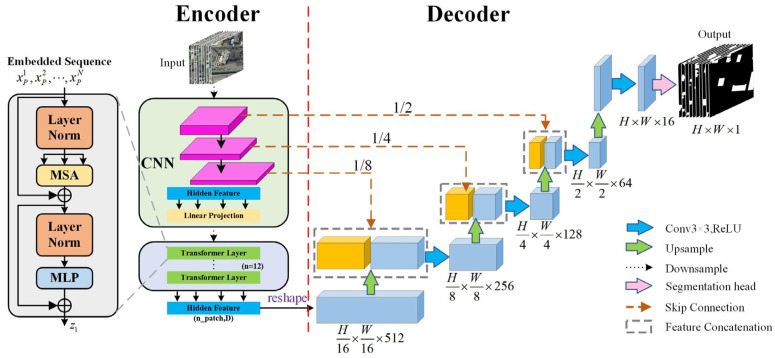
Architecture of the TransUNet network.

**Figure 3:**
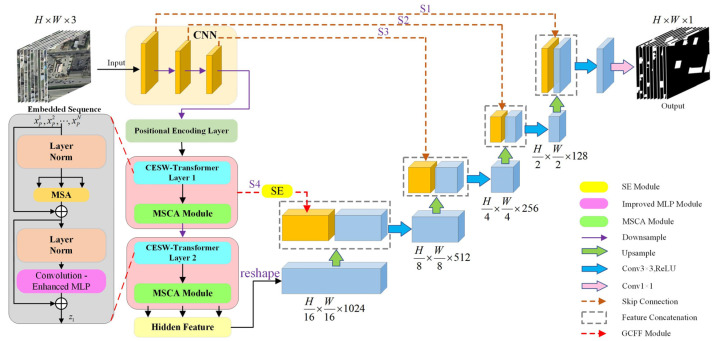
Architecture of the improved CESW-TransUNet network.

By jointly optimizing local perception and global modeling, this architecture effectively mitigates the loss of edge details in complex building structures, providing robust support for photovoltaic resource assessment. The baseline TransUNet framework is illustrated in Figure 2, while the overall structure of the improved model is shown in Figure 3.

#### 2.2.1. Convolution-Enhanced Swin Transformer Module

**Figure 4:**
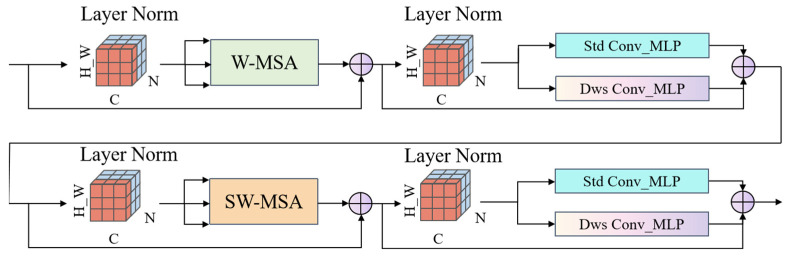
Architecture of the Convolution-Enhanced Swin Block (CESW-Block), which incorporates a dual-branch design of standard and depthwise separable convolutions within the MLP layer to enhance local feature extraction and significantly reduce computational overhead.

**Figure 5:**
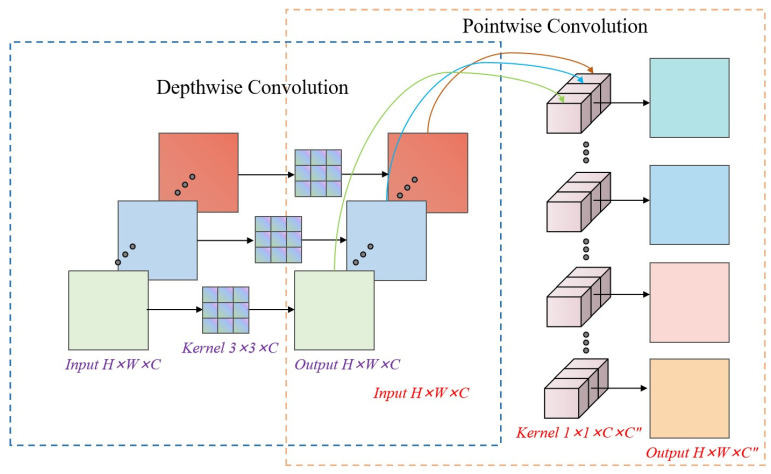
Architecture of the depthwise separable convolution (DWS Conv) module, which factorizes a standard convolution into a depthwise and a pointwise convolution to dramatically reduce computational complexity and model parameters while maintaining representative capacity.

To address the high computational complexity and limited local feature extraction capability associated with the fully connected (MLP) layers in the conventional Swin Transformer [17], we propose a Convolution-Enhanced Swin Block (CESW-Block), as illustrated in Figure 4 and Figure 5.

This module adopts a dual-branch parallel architecture within the MLP layer: one branch utilizes a standard 3 × 3 convolutional layer to preserve the spatial locality prior and effectively enhance edge feature extraction through a fixed receptive field; the other branch introduces a depthwise separable convolution, which, via a spatial-channel decoupling strategy, reduces the computational complexity from O(n^2^k^2^d^2^) to O(n^2^k^2^ + n^2^d^2^). This design substantially improves computational efficiency while maintaining feature extraction performance, thus enabling lightweight model architecture.

After parallel execution, the outputs from the standard convolution and depthwise separable convolution (DWS Conv) branches are fused through element-wise addition, followed by GELU activation, as shown in Equation (1):

(1) $$F_{CESW}=GELU(F_{std}+F_{dws})$$

where *F_std_* and *F_dws_* denote the outputs of the standard convolution and DWS Conv branches, respectively, and GELU represents the Gaussian Error Linear Unit activation.

By incorporating DWS Conv branches with different dilation rates, the module is able to capture both local edge and global structural features. Finally, a residual connection is employed to mitigate the vanishing gradient problem commonly observed in deep Transformer architectures.

#### 2.2.2. Multi-Scale Channel Attention Module (MSCA)

Although the CESW-Block enhances feature extraction capability, the inherent multi-scale characteristics of buildings and channel redundancy continue to limit segmentation accuracy. To address these challenges, a Multi-Scale Channel Attention (MSCA) module [18] is integrated at the back end of the CESW-Block.

**Figure 6:**
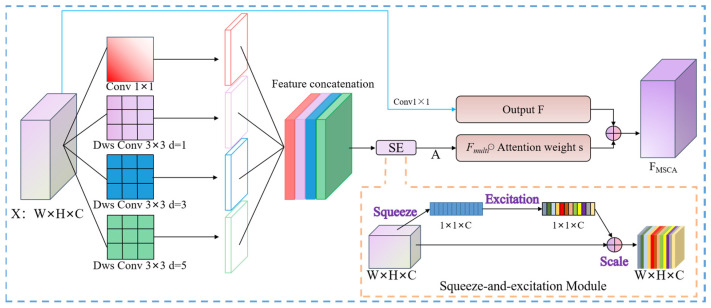
Architecture of the Multi-Scale Channel Attention (MSCA) module, which employs parallel convolutional branches with varying receptive fields to capture multi-scale features and a squeeze-excitation mechanism to recalibrate channel-wise importance, thereby enhancing the segmentation of buildings across different scales.

This module employs parallel branches with varying receptive fields to adaptively capture building features across multiple scales, while the channel attention mechanism is leveraged to enhance key feature selection and reduce channel redundancy. The architecture of the MSCA module is illustrated in Figure 6.

This module adopts a multi-branch collaborative architecture. Initially, four parallel convolutional branches with distinct receptive fields are employed to extract multi-scale spatial features. These feature maps are then concatenated along the channel dimension to form a hierarchical feature representation.

Subsequently, a squeeze-and-excitation (SE) mechanism is introduced to adaptively recalibrate channel-wise feature responses, thereby suppressing irrelevant features. Finally, the refined feature map is produced through feature weighting and a residual connection strategy. The overall computation is formulated as follows:

(2) $$F_{multi}=Concat{\left(Branch_{i}(X)\right)}_{i=1}^{4}$$

(3) $$z[c]=\frac{1}{H\times W}\sum_{h=1}^{H}\sum_{w=1}^{W}F_{multi}[c,h,w]$$

(4) $$s=\sigma (W_{2}(\mathrm{ReLU}(W_{1}(z))))$$

(5) $$F_{\mathrm{MSCA}}=F_{\mathrm{multi}}⊙s+\mathrm{Align}(F)$$

In this formulation, *F_multi_* denotes the multi-scale feature representation, *z* represents the output of global average pooling, *W*_1_ and *W*_2_ are the weight matrices of the fully connected layers, *σ* indicates the sigmoid activation function, ⊙ denotes element-wise multiplication, and Align refers to the channel alignment operation implemented via a 1 × 1 convolution.

By jointly optimizing multi-scale local perception and global channel attention, this design addresses the limited adaptability of conventional single-branch attention mechanisms in cross-scale building feature extraction. In addition, it enhances the robustness of the model against noise interference in high-resolution imagery.

#### 2.2.3. Global-Guided Cross-Level Feature Fusion (GCFF)

To address the blurring of semantic feature boundaries at higher layers and the lack of global guidance in skip connections inherent to traditional TransUNet architectures, we propose a Global-guided Cross-level Feature Fusion (GCFF) mechanism.

In this approach, the sequential features Z output by the CESW-Block in the intermediate layer are first processed and reshaped into a spatial format. Convolutional operations are then applied to align the feature dimensions, resulting in the generation of S_4_, which is injected into the decoder. By leveraging high-level semantic information to guide the upsampling of low-level features, this mechanism mitigates the loss of edge details in building structures and improves sensitivity in detecting small-scale buildings. The detailed computational process is as follows:

(6) $$Z=CESWBlock_{1}(\;\underset{ResNet\;layer_{2}}{\underset{︸}{F_{CNN}^{3}}})$$

(7) $$S_{4}=Reshape(Z)\to S_{4}\in {\mathbb{R}}^{B\times 512\times \frac{H}{8}\times \frac{W}{8}}$$

(8) $$A=\sigma (Conv_{1\times 1}(Concat(UpSample(F_{deep}),S_{4})))$$

(9) $$Y_{1}=UpSample(F_{deep})⊙A+S_{4}⊙(1-A)$$

In this formulation, *Z* denotes the enhanced feature representation produced by the CESW-Block; *S*_4_ represents the feature map obtained after dimension reshaping; *A* corresponds to the global attention weights; and *Y*_1_ indicates the cross-level fused features.

### 2.3. Evaluation Metrics

In this study, the confusion matrix serves as the core analytical framework for a comprehensive quantitative evaluation of network model performance. Five metrics are employed as evaluation indices: overall accuracy (Pa), precision (Pr), intersection over union (IoU), recall (Re), and the F1 score. The calculation formulas for these five metrics are provided in Table 1.

The evaluation metrics are defined as follows, where TP (true positive) refers to the number of building regions that are correctly predicted as buildings by the model; FP (false positive) denotes the number of non-building regions that are incorrectly predicted as buildings; FN (false negative) represents the number of building regions that are incorrectly predicted as non-buildings; and TN (true negative) indicates the number of non-building regions that are correctly predicted as non-buildings.

### 2.4. Rooftop Photovoltaic Resource Potential Assessment: Theoretical Framework

#### 2.4.1. Fundamental Theory of Solar Radiation and Model Development

In rooftop photovoltaic (PV) resource assessment, this study comprehensively considers two primary parameters: the available installation area for PV modules and the incident solar radiation. Based on a regional solar radiation model [19], a dedicated rooftop radiation calculation model is developed. This model quantifies hourly direct, diffuse, and reflected radiation components by incorporating parameters such as latitude and rooftop geometry. Among these, direct radiation contributes the most, followed by diffuse radiation, while reflected radiation is typically negligible. The model expression is presented in Equation (10):

(10) $$H_{A}=I_{s}+I_{d}+I_{r}$$

where *H_A_* represents the total rooftop solar radiation; *I_s_* denotes the direct radiation component; *I_d_* is the diffuse radiation component; *I_r_* is the reflected radiation component; the radiation unit is kWh/m^2^.

#### 2.4.2. Calculation of Effective Rooftop Area

(1) Basic Area Calculation

In the assessment of distributed rooftop photovoltaic (PV) potential, the primary task is to accurately determine the effective and available rooftop area of buildings. Based on the fusion results of multiple model predictions, this study utilizes Python (version 3.9.13) programming to perform pixel-level area inversion. By analyzing the spatial resolution of raster images and combining it with the number of pixels corresponding to rooftop regions, the actual area of the target region can be computed. The specific formula is provided in Equation (11):

(11) $$A=n\times r^{2}$$

where *A* is the total predicted area of the target region; *n* is the number of pixels; *r* is the actual ground size represented by each pixel.

(2) Correction of Effective Rooftop Area

On this basis, it is necessary to comprehensively account for the effects of inter-building shadowing, the area occupied by rooftop fixtures, and regions unsuitable for photovoltaic installation. The formula for calculating the corrected effective rooftop area is given in Equation (12):

(12) $$G=\left(A-U\right)\times B_{1}\times B_{2}$$

where *G* is the effective rooftop area; *A* is the rooftop area segmented by the model; *B*_1_ is the correction factor for building shadow occlusion; *B*_2_ is the correction factor for rooftop fixtures; *U* is the area unsuitable for rooftop photovoltaic installation.

The rooftop fixture ratio coefficient (*B*_2_) is determined through statistical sampling of high-resolution remote sensing imagery within the target area. The specific procedure includes: (1) extracting rooftop fixture features based on visible and near-infrared bands; (2) employing a random sampling method to manually verify no less than 5% of the total number of buildings; (3) calculating the mean fixture ratio via area-weighted averaging. For regions unsuitable for photovoltaic installation, this study adopts a combination of morphological segmentation and area thresholding to identify and exclude isolated regions smaller than 2 m^2^.

Regarding shadow occlusion between buildings, this essentially represents the spatiotemporal loss of solar radiation energy. Sunlight is readily blocked by tall structures during its path, resulting in insufficient illumination for certain areas of lower rooftops and, consequently, reduced photovoltaic system efficiency.

To accurately quantify the shadow effect, this study proposes a radiation-to-area equivalence conversion method: the ratio of the annual cumulative solar radiation under shadow-free conditions (*E_y_*_0_) to that under actual shadow conditions (*E_y_*_1_) is defined as the static effective area reduction coefficient (*B*_1_).

**Figure 7:**
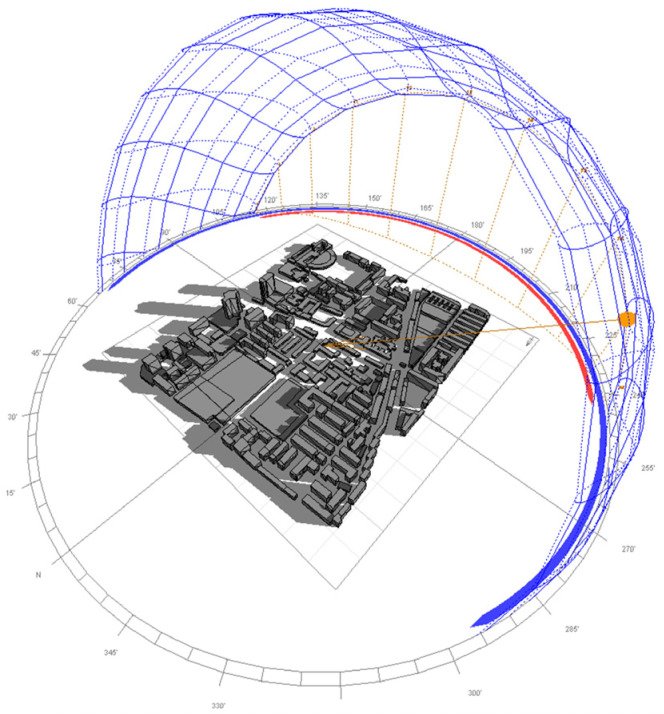
Conceptual diagram of inter-building shading effects in a representative urban area, illustrating how building geometry and solar position jointly influence rooftop solar access.

To model dynamic shading effects, a physics-based simulation was implemented. Building rooftop contours, precisely extracted via our semantic segmentation network, were integrated with elevation data derived from the Weijing Map Platform. Building heights were estimated based on floor count information from the platform, using a standard height of 2.8 m per story as stipulated in China’s Residential Design Code. This approach offers a practical and regionally appropriate means of constructing large-scale 3D urban models for solar analysis in contexts where LiDAR data is unavailable. The resulting 3D model was processed in Ecotect Analysis to perform annual solar ray-tracing simulations. Shadow distributions were calculated hourly to quantify spatiotemporal occlusion patterns. Figure 7 illustrates a conceptual representation of inter-building shading within a representative area of the study site.

**Figure 8:**
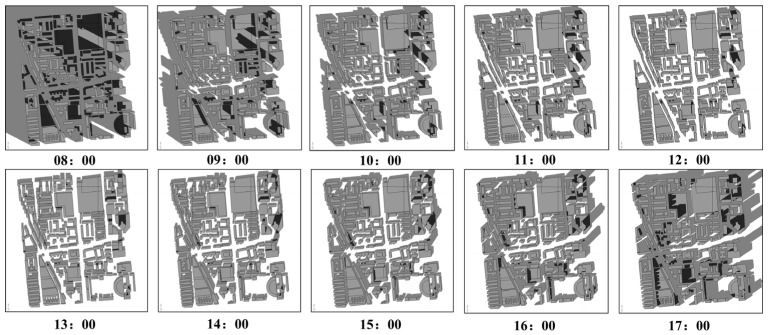
Simulated rooftop shadow patterns at different times for a representative subset of the study area.

To visualize these temporal variations, Figure 8 displays hourly shadow occlusion patterns observed throughout a representative day.

**Figure 9:**
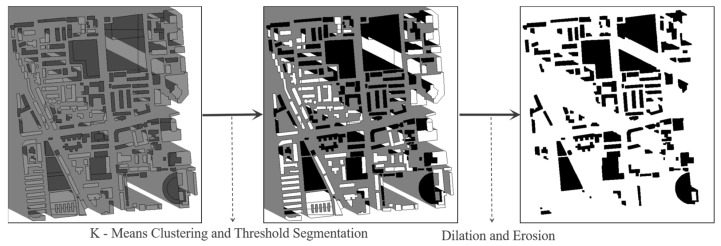
Exemplary result of rooftop shadow segmentation using K-means clustering and morphological processing, which effectively isolates and classifies shadow pixels specifically belonging to building rooftops at a selected time point.

To effectively distinguish rooftop shadows from ground shadows across different time intervals, a combination of K-means clustering and thresholding was employed. This approach classifies image pixels into three distinct categories: ground shadows, rooftop shadows, and non-shadowed rooftop areas. Subsequently, morphological processing operations (e.g., closing, opening) were applied to refine the segmentation, specifically isolating contiguous pixel regions belonging to rooftop shadows. A representative result of this segmentation process is illustrated in Figure 9.

For the calculation of solar radiation under shadow-free conditions, the annual total solar radiation received by the target area under ideal circumstances is determined based on the dynamic variation data of solar radiation throughout the year. The calculation formula is provided in Equation (13):

(13) $$E_{y0}=\sum_{m=1}^{12}\left[\sum_{d=1}^{n}\left(\sum_{h=1}^{24}G_{0}\times H_{Ah}\right)\right]$$

where *E*_*y*0_ is the total daily solar radiation on building rooftops under shadow-free conditions; *G*_0_ is the total rooftop area without shadows for the entire day; *H_Ah_* is the solar radiation in the target area at each hourly interval throughout the day (h = 1, 2, 3, …, 24, hour); n is the number of days in each month (n = 1, 2, 3, …, 31, day); j is the month index (j = 1, 2, 3, …, 12, month).

Based on this, the annual total rooftop solar radiation, accounting for shadow occlusion between buildings, is calculated. The calculation formula is presented in Equation (14):

(14) $$E_{y1}=\sum_{m=1}^{12}\left[\sum_{d=1}^{n}\left(\sum_{h=1}^{24}G_{i}\times H_{Ah}\right)\right]$$

where *E*_*y*1_ is the total annual rooftop solar radiation considering shadow effects; *G_i_* is the rooftop area without shadow at each hourly interval throughout the day.

Finally, based on the solar radiation values under both shadowed and shadow-free conditions, the corrected effective rooftop area reduction coefficient for the target area, B1, is determined. The calculation formula is given in Equation (15):

(15) $$B_{1}=\frac{E_{y1}}{E_{y0}}$$

This conversion preserves the spatiotemporal variability of shadows (e.g., seasonal and hourly changes), while simultaneously simplifying the core input parameters required for PVsyst photovoltaic array design—only the corrected effective rooftop area is needed.

#### 2.4.3. Calculation Method for Rooftop Photovoltaic Power Generation

After obtaining the corrected effective rooftop area (G), this study utilizes PVsyst software (version 7.4) to assess photovoltaic generation potential. The software uses the effective area as a core input parameter and, through its built-in algorithms, automatically completes PV module selection, array layout optimization, and the coupling of micro-scale shading effects between modules to achieve precise simulation.

The calculation procedure consists of three main steps. First, based on the effective rooftop area and the physical dimensions of the PV modules, the maximum number of deployable PV modules is calculated according to Equation (16):

(16) $$N=\frac{G}{S_{module}}$$

where *N* is the number of deployable PV modules (units); *S_module_* is the area of a single module (m^2^ per unit).

After determining the number of modules, the total installed capacity of the photovoltaic system P_AZ_ is calculated by combining the rated power of a single module and the system adaptation coefficient, as given in Equation (17):

(17) $$P_{AZ}=N\times P_{module}\times C_{basic}$$

where *P_module_* is the rated power of a single module (kWp per unit); *C_basic_* is the system adaptation coefficient.

After determining the total installed capacity, the grid-connected electricity generation (*E_P_*) is calculated using the built-in simulation modules of the PVsyst software (version 7.4). The software relies on a physical model of the photovoltaic system and automatically integrates all relevant factors, including local effective irradiance, module temperature characteristics, inverter efficiency, and electrical losses. The core calculation logic corresponds to Equation (18):

(18) $$E_{p}=H_{eff}\times P_{AZ}\times \eta_{sys}$$

where *E_P_* is the grid-connected electricity generation (kWh); *H_eff_* is the effective irradiance (kWh/m^2^); *η*_sys_ is the overall system efficiency.

### 2.5. Post-Processing in Semantic Segmentation Tasks

#### 2.5.1. Overview of the Residual-Based Fusion Strategy

In deep learning-based semantic segmentation tasks, single network models are often affected by learning bias and overfitting, leading to deviations in their prediction results [20,21,22]. With the widespread improvements and diversification of semantic segmentation models, the fusion of multi-model prediction results has become a common strategy. However, traditional majority voting fusion methods face issues such as weight dilution and interference from low-quality predictions, which negatively impact the overall fusion accuracy. To address these challenges, this study proposes a residual fusion strategy based on ensemble learning principles, aimed at improving the overall accuracy and robustness of multi-model semantic segmentation [23]. This approach innovatively transforms the full-image fusion problem into patch-level local decisions: The image is first divided into several non-overlapping local patches, and the predictions of the optimal model with high accuracy are used as a baseline. Auxiliary model patches that highly align with the optimal model’s predictions in local regions are selected, and only the reliable local pixel information filtered by this consistency screening is incorporated into the fusion process. Compared to traditional full-image majority voting methods, this strategy significantly reduces the interference from low-quality models and effectively avoids the dilution of the dominant model’s weight and the accumulation of noise.

To further enhance the advantages of multi-model fusion, the proposed residual fusion mechanism does not adjust all predicted pixels of the optimal model uniformly. Instead, it focuses on local pixels where there are discrepancies between the optimal model and auxiliary models. For these regions, if two or more auxiliary models agree, their voting results directly replace the prediction value of the optimal model for the corresponding pixel, achieving high-confidence local corrections. This strategy effectively utilizes the complementary information between models while retaining the high-accuracy performance of the optimal model in most areas, thereby balancing segmentation precision and robustness.

Theoretically, this method combines the bias-variance decomposition framework in ensemble learning, fully leveraging the low-bias characteristic of the optimal model and the complementary errors and weak correlations between auxiliary models, thus effectively reducing overall generalization error and improving decision stability. This strategy is both simple and efficient in engineering implementation, providing an innovative and practical fusion paradigm for multi-model semantic segmentation tasks.

#### 2.5.2. Fusion Procedure

**Figure 10:**
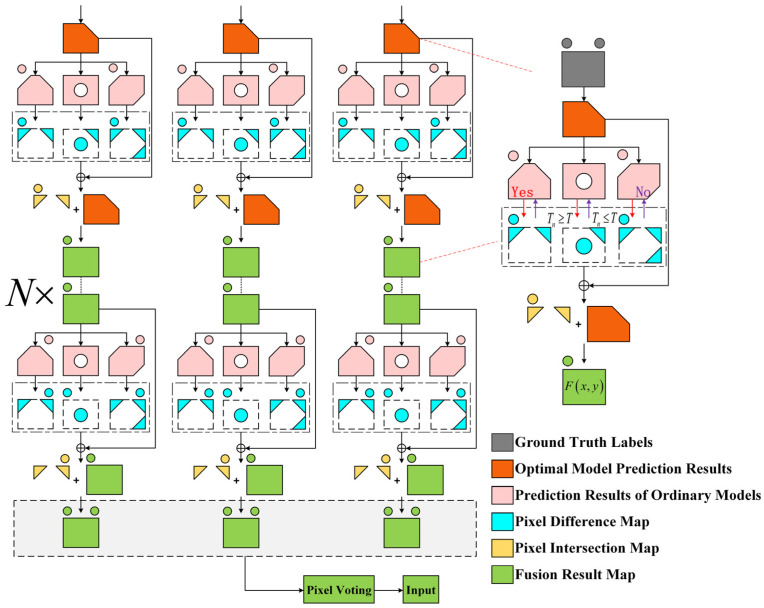
Schematic of the patch-wise residual fusion strategy, which employs a local consistency screening mechanism to selectively integrate predictions from multiple models, thereby reducing generalization error by leveraging the low-bias of the optimal model and error diversity of auxiliary models.

This study selected 12 pre-trained semantic segmentation models and chose the optimal model based on a weighted composite score of five evaluation metrics: IoU, F1-score, Precision, Recall, and Accuracy. The specific process of the overall strategy is shown in Figure 10.

First, the composite performance score *S_j_* of the *j*-th model is defined, and the three models with the highest *S_j_* values are selected as the baseline group {A1, A2, A3}. Next, pixel-wise majority voting is applied to the prediction results of the baseline models to generate the global synthesized pseudo-label *A_syn_*, and the calculation process is as follows:

(19) $$S_{j}=\alpha \cdot IoU_{j}+\beta \cdot F1_{j}+\gamma \cdot Pre_{j}+\delta \cdot Re_{j}+\epsilon \cdot Acc_{j}$$

(20) $$A_{syn}(x,y)=I(\sum_{m=1}^{3}A_{m}\left(x,y\right)\geq 2)$$

In the formula, the *α*, *β*, *γ*, *δ*, and *ε* represent the weighting coefficients, which satisfy the condition that their sum equals 1; *I*(·) is the indicator function (which takes the value 1 when the condition is true, and 0 otherwise), and (x, y) denotes the pixel coordinates.

The input image to be predicted is divided into P × P patches {*P_k_*} (with k representing the patch index), synchronized with the synthesized pseudo-label *A_syn_*. Then, for each of the 9 non-baseline models {B_n_} (*n* = 1, 2, …, 9), the weighted proximity between the prediction for each patch k and the corresponding pseudo-label patch *A_syn_*^(*k*)^ is calculated. Next, the selection threshold for each patch *B_n_*^(*k*)^ is computed, which is the mean proximity across all 9 models. The calculation process is as follows:

(21) $$T_{n}^{\left(k\right)}=\alpha \cdot IoU_{n}^{\left(k\right)}+\beta \cdot F1_{n}^{\left(k\right)}+\gamma \cdot Pre_{n}^{\left(k\right)}+\delta \cdot Re_{n}^{\left(k\right)}+\epsilon \cdot Acc_{n}^{\left(k\right)}$$

(22) $$T^{\left(k\right)}=\frac{1}{9}\sum_{n=1}^{9}T_{n}^{\left(k\right)}$$

where *T_n_*^(*k*)^ is the weighted proximity between the prediction of the *n*-th model on patch k and the corresponding patch of the synthetic pseudo-label.

**Figure 11:**
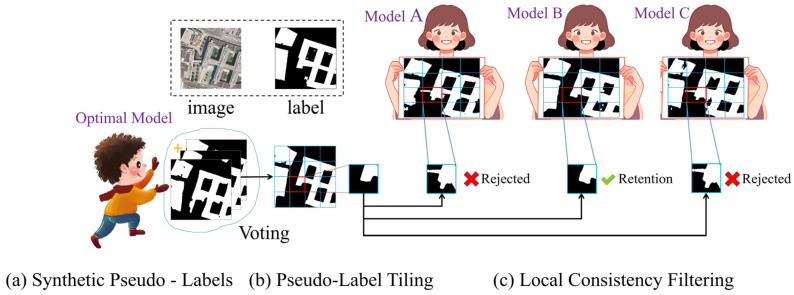
Schematic of the Local Consistency Screening mechanism, which filters out unreliable model predictions within local patches by applying a threshold-based selection criterion to ensure robustness in the fusion process.

When the proximity *T_n_*^(*k*)^ of a model *B_n_*^(*k*)^ within the patch P_k_ is less than or equal to the threshold *T*^(*k*)^, the prediction result of this model is considered unreliable and will not participate in the subsequent voting fusion. This step effectively eliminates predictions that differ significantly from the synthesized label, ensuring local consistency in the voting fusion process. A schematic diagram of the patch selection process is shown in Figure 11.

Subsequently, the model results that passed the screening are randomly assigned to three groups {G_1_, G_2_, G_3_}, corresponding to the baseline models {A_1_, A_2_, A_3_} respectively. Then, the differing pixel regions between the models in each group and the baseline models are marked, with the calculation formula as follows:

(23) $$D_{m,n}^{\left(k\right)}(x,y)=I({B_{n}}^{\left(k\right)}(x,y)\neq {A_{m}}^{\left(k\right)}(x,y))$$

If the predictions of two or more models in the differing region are consistent, the baseline model is corrected using the mode; otherwise, the original prediction is retained:

(24) $${A_{m}}^{\left(k\right)}{\left(x,y\right)}_{new}=\{\begin{array}{ll} Mode(\{{B_{n}}^{\left(k\right)}(x,y)|n\in G_{m},D_{m,n}^{\left(k\right)}(x,y)=1\}) & if\sum_{n\in G_{m}}D_{m,n}^{\left(k\right)}(x,y)\geq 2\\ {A_{m}}^{\left(k\right)}(x,y) & otherwise \end{array}$$

where Mode(·) denotes taking the mode of the set.

Finally, majority voting is applied to the three groups of corrected results, using the three corrected baseline models (A_1_, A_2_, A_3_) to generate the final result. In this process, each corrected baseline model (A_1_, A_2_, A_3_), after undergoing local consistency screening and correction, undergoes another round of majority voting to determine the final result. The purpose of this approach is to integrate multiple corrected optimal models, further improving accuracy and robustness, reducing errors from a single model, and ensuring more stable and reliable fusion results. The calculation formula is as follows:

(25) $$F_{final}(x,y)=I(\sum_{m=1}^{3}{A_{m}}^{\left(k\right)}{\left(x,y\right)}_{new}\geq 2)$$

## 3. Results

### 3.1. Experimental Environment Configuration

The experiments were conducted on a workstation equipped with an Intel 12th Generation Core i7-12700F processor, 32 GB RAM, and an NVIDIA GeForce RTX 4070 GPU featuring 12 GB of dedicated memory and 16 GB of shared memory. The operating system was Windows 10.

All model training and implementation were performed using the Python (version 3.9.13) programming language within the PyCharm (version 2023.3.6) integrated development environment. All convolutional neural network models were constructed based on the PyTorch framework (version 2.1.0), and geographic information processing was supported by ArcGIS Pro (version 3.4.0).

### 3.2. Dataset Processing and Training

The INRIA Aerial Image Labeling Dataset was utilized in this study. All images were standardized by cropping into 512 × 512-pixel tiles, yielding 13,238 valid samples. These samples were divided into training, validation, and test sets at an 8:1:1 ratio. All benchmark metrics, including IoU and F1-score, were computed based on this split to ensure reproducibility.

To improve generalization to local architectural features—such as sloped roofs and dense building clusters in the Kunming University Town area—a custom dataset of 3663 annotated samples was created from Google Earth imagery at 0.5 m resolution. This custom set was not used in the initial benchmark evaluation but was incorporated in a fine-tuning stage, where it was blended with the INRIA data to adapt the pre-trained models to regional roof characteristics. The hyperparameters employed for model training are provided in Table 2.

Given the widespread application of semantic segmentation models, this study selects several state-of-the-art architectures for performance comparison and as the foundation for subsequent multi-model prediction fusion. The models considered include U-Net [24], DeepLabv3+ [25], PSPNet [26], FCN [27], UPerNet [28], MobileNet [29], DNLNet [30], APCNet [31], SegFormer [32], TransUNet [16], Swin-T [17], as well as the improved model proposed in this study.

### 3.3. Training Results

**Figure 12:**
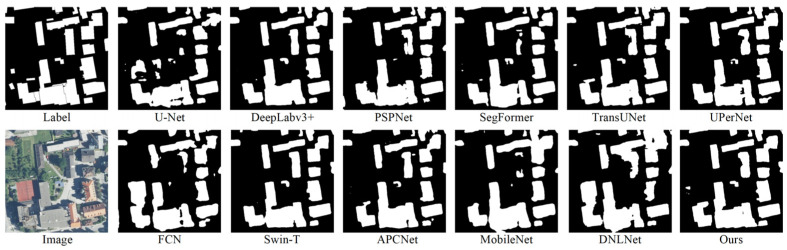
Comparison of model prediction results on the INRIA dataset.

**Figure 13:**
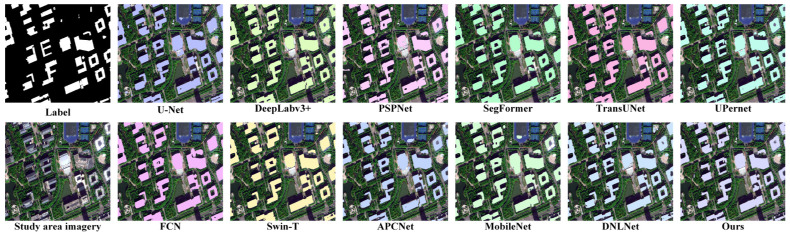
Comparison of model prediction results on images from the study area.

A comprehensive performance evaluation was conducted for the proposed CESW-TransUNet model, with comparative experiments against mainstream semantic segmentation models on both the INRIA dataset and the study area (qualitative results are presented in Figure 12 and Figure 13).

Traditional CNN-based methods, such as U-Net and FCN, exhibit suboptimal performance in building boundary segmentation, often resulting in over-segmentation or under-segmentation and consequently producing blurred contours—an issue that is further amplified in areas with complex rooftop morphologies.

Transformer-based models, including SegFormer, TransUNet, and Swin-T, are capable of modeling long-range dependencies, but are associated with higher computational costs and a tendency to miss small objects. This leads to reduced effectiveness in detecting small, scattered rooftops within the study area.

In contrast, the proposed model achieves significantly improved building rooftop prediction accuracy while maintaining efficient training. It demonstrates clear boundary segmentation on the INRIA dataset and maintains precise contour recognition in real-world scenarios, substantially reducing the omission rate for small rooftops.

The results from both the open-source dataset and the complex study area validate the strong adaptability of the model for rooftop segmentation tasks, ensuring robust generalization to generic scenarios while being precisely tailored to the practical needs of the study area. This provides a more reliable technical foundation for the engineering application of building rooftop segmentation.

A comprehensive evaluation of both performance and efficiency for all models was conducted through quantitative experiments, as summarized in Table 3.

In terms of segmentation accuracy, the proposed CESW-TransUNet outperformed all baseline models, achieving 78.50% IoU and 88.36% F1 score, representing improvements of 2.27 and 2.01 percentage points, respectively, over the baseline TransUNet. The model also demonstrated balanced precision (88.60%) and recall (88.12%) values.

From an efficiency optimization perspective, the CESW-TransUNet incorporates lightweight design strategies such as reconstructing local feature extraction paths with depthwise separable convolutions and replacing the standard Transformer MLP with a dual-branch structure. These improvements resulted in a substantial reduction in computational overhead: the number of parameters (90.89M) is reduced by 26.6% compared to TransUNet (123.84M); computational cost (67.69 GFLOPS) is reduced by 21.0% relative to TransUNet (85.67 GFLOPS); and inference speed (32.69 FPS) is improved by 24.8% over TransUNet (26.18 FPS).

In horizontal comparison, the proposed model delivers SOTA segmentation accuracy while significantly surpassing other high-performance architectures in computational efficiency (e.g., Swin-T achieves 65.15 GFLOPS and 23.04 FPS), and it avoids sacrificing accuracy for speed (e.g., MobileNet achieves only 83.47% F1 score). The experimental results demonstrate that the proposed lightweight strategies effectively balance accuracy and efficiency in complex segmentation tasks, offering an optimal solution for photovoltaic engineering applications in resource-constrained environments.

To evaluate the contribution of each module to the overall model performance, ablation experiments were conducted on the INRIA validation set, as summarized in Table 4. The baseline model, which does not incorporate any additional modules, serves as the reference. Subsequently, the SW-Block, CESW-Block, MSCA, and GCFF modules were individually or jointly added to the model, and the performance under each configuration was compared.

The results indicate that adding any single module leads to performance improvements over the baseline, with the CESW-Block yielding the most significant gains in pixel accuracy (Pa) and IoU—improving by 1.35% and 1.93%, respectively. The SW-Block, MSCA, and GCFF also contributed varying degrees of enhancement. Notably, the full model, which integrates all modules, achieved the highest overall performance, reaching peak values in all metrics (Pa 95.47%, IoU 78.50%, and F1 score 88.36%), and substantially outperforming any single-module configuration.

These ablation results clearly demonstrate the effectiveness of each module and highlight the synergistic benefits of their joint integration for comprehensive model performance enhancement.

### 3.4. Analysis of Fusion Results

As shown in Table 5, among the baseline models, CESW-TransUNet achieved the best single-model performance (IoU 78.50%, F1 88.36%). The full-range majority voting fusion exhibited significant fluctuation in the five-fold generalization validation (with 80% of samples used to construct the fusion rules and 20% reserved for testing), with an IoU of 77.56% ± 0.82 and an F1 score of 87.83% ± 0.47, which was lower than all single models. In contrast, the residual fusion strategy proposed in this study resulted in improved accuracy under the same validation conditions: IoU reached 79.85% ± 0.18 (with a 78% reduction in standard deviation), and F1 was 89.28% ± 0.12. This strategy implements local reliability screening through the patch-wise residual fusion mechanism, which suppresses error propagation and reduces the fluctuation range of key metrics to 22% of that of the full-range fusion, demonstrating its strong adaptability to spatial heterogeneity.

### 3.5. Application and Evaluation in the Study Area

#### 3.5.1. Overview of the Study Area

**Figure 14:**
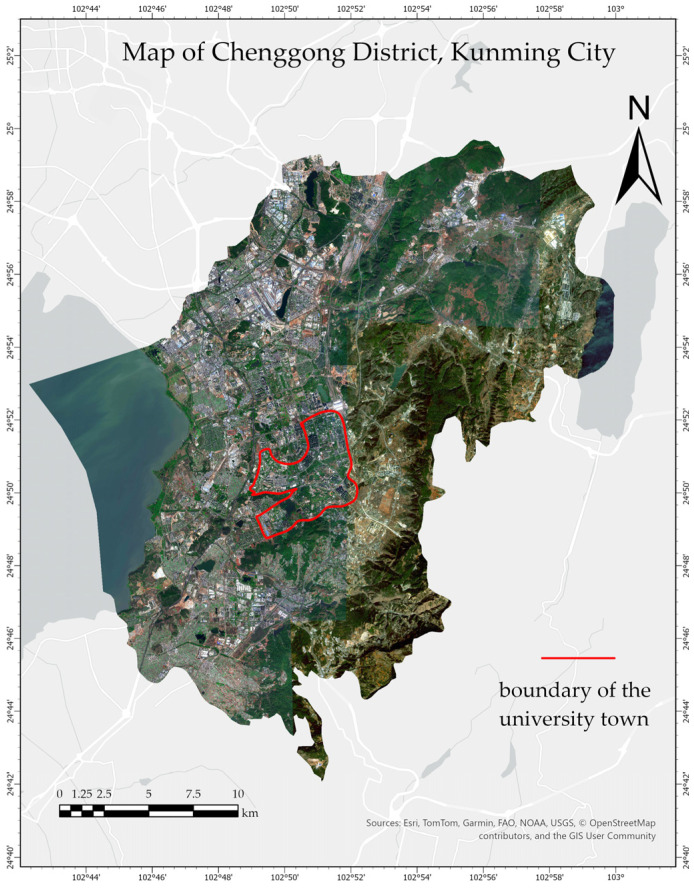
Map of the study area.

This study focuses on the newly constructed university campuses in Kunming City, located at approximately 102.84° E and 24.85° N. The region enjoys long sunshine hours and abundant solar irradiance, making it one of the areas rich in solar energy resources. The modern architectural design and infrastructure of these new campuses facilitate the installation of photovoltaic (PV) projects, making the area suitable for both the development of the PV industry and educational promotion. An overview of the study area is provided in Figure 14.

#### 3.5.2. Visualization Analysis of Residual Fusion

**Figure 15:**
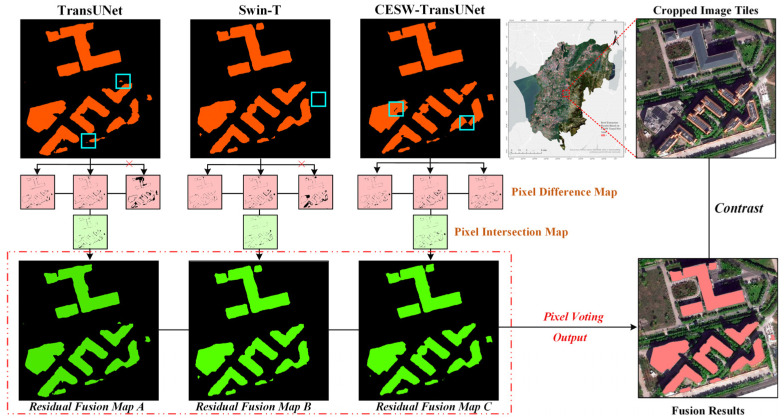
Refined rooftop segmentation results through the proposed residual fusion strategy.

Twelve sets of semantic segmentation model predictions were selected for fusion in this study, aiming to leverage the learning diversity among different models to improve the overall prediction accuracy. The results of the fusion process are illustrated in Figure 15.

First, the three best-performing models—TransUNet, SWin-T, and the model proposed in this study—were selected from the twelve candidate models as reference models for fusion. The fused results not only provide a more precise delineation of building boundaries but also effectively address local prediction omissions that may occur in individual models. As a result, the final segmentation outputs more closely resemble the actual remote sensing imagery, with notable improvements in both overall structure and fine details.

Overall, the hierarchical multi-model residual fusion strategy effectively mitigates local prediction errors inherent in single-model approaches, enhances the accuracy of rooftop pixel prediction, and demonstrates significant value for semantic segmentation post-processing.

#### 3.5.3. Comprehensive Assessment of Photovoltaic Potential in the Study Area

**Figure 16:**
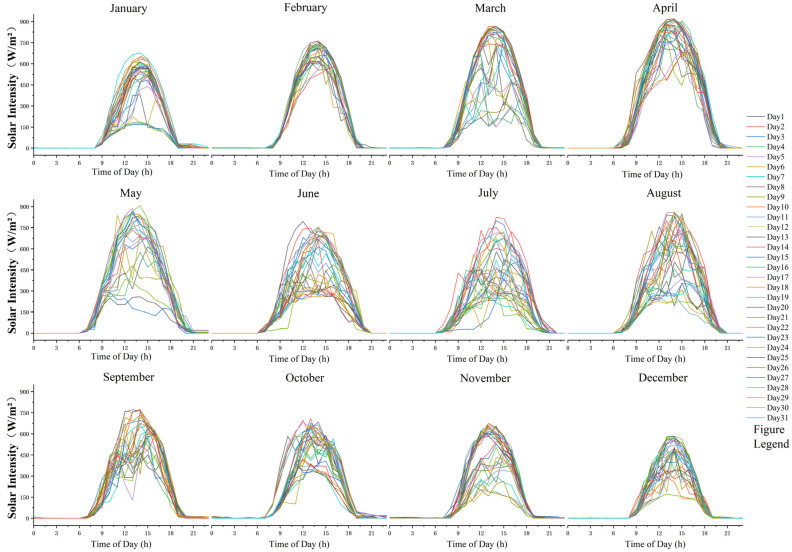
Diurnal variation curves of solar radiation intensity in the target area throughout the year.

In this study, high spatiotemporal resolution solar radiation data for Chenggong District, Kunming, covering the entirety of the year 2024, were obtained from the Environmental Meteorological Data Service Platform (http://eia-data.com/surf_chn_mul_hor_lite/ (accessed on 18 September 2025)). As shown in Figure 16, solar radiation intensity exhibits a pronounced diurnal variation, with peak values observed during the midday period (12:00–14:00). Analysis of interannual variation indicates that the study area possesses abundant solar energy resources, with a total annual solar radiation reaching 1572 kWh/m^2^. Notably, radiation intensity is highest in spring (March to May), highlighting the significant potential for photovoltaic development in this region.

**Figure 17:**
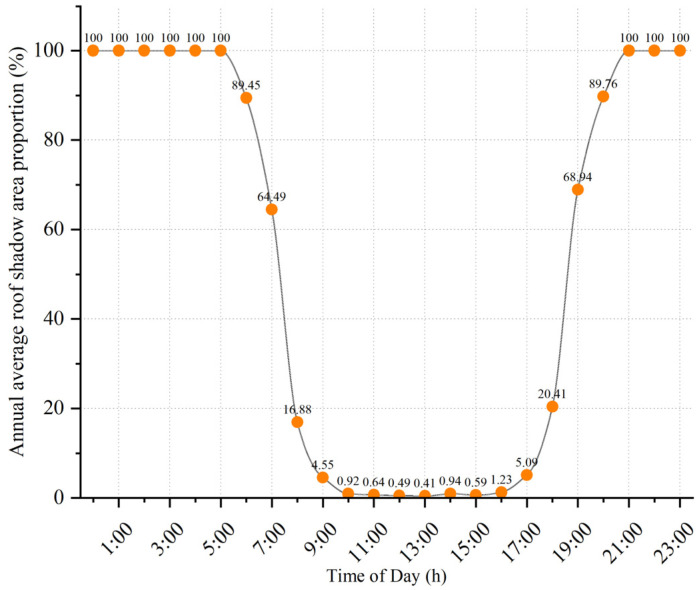
Annual average rooftop shadow proportion in the target area.

Subsequently, based on the fusion of multi-model prediction results, the rooftop shadow area for each hourly interval throughout the year was statistically analyzed for the target region. To intuitively assess the impact of shadows on rooftop solar radiation reception, the annual spatiotemporal distribution of average rooftop shadows was further calculated, as illustrated in Figure 17. The diurnal variation curve of rooftop shadow proportion displays a characteristic U-shape, with the minimum shadow coverage occurring at 13:00, when the solar elevation angle reaches its maximum. At other times, especially during early morning and late afternoon, the shadow proportion is relatively higher, resulting in less rooftop solar irradiance.

To evaluate the annual greenhouse gas emissions avoided by rooftop photovoltaic (PV) power generation compared to conventional coal-fired power, this study calculated carbon emission reductions based on the “Methodology for Renewable Energy Grid-Connected Power Generation” (CM-001-V02, Second Edition). The combined margin CO_2_ emission factor, *EF_grid,CM,y_*, is calculated as shown in Equation (26):

(26) $$EF_{grid,CM,y}=EF_{grid,OM,y}\times W_{OM}+EF_{grid,BM,y}\times W_{BM}$$

where *EF_grid,OM,y_* and *EF__grid,BM,y_* represent the operating margin and build margin emission factors for year y (tCO_2_/MWh), respectively, based on the most recent regional grid baseline emission factors published by the National Development and Reform Commission (NDRC) of China. W_OM_ and W_BM_ denote the weights of the operating margin and build margin emission factors, respectively.

Based on the most recent regional grid baseline emission factors published by the Ministry of Ecology and Environment of China, the combined margin CO_2_ emission factor for the study area was calculated to be 0.6299 tCO_2_/MWh. The corresponding annual carbon emission reduction can then be obtained, as expressed in Equation (27):

(27) $$BE_{y}=EG_{pj,y}\times EF_{grid,CM,y}$$

where *BE_y_* denotes the annual carbon emission reduction, and *EG_pj,y_* represents the annual electricity generation.

After comprehensively accounting for all factors influencing rooftop availability—including structural constraints, shading effects, and rooftop infrastructure—the effective rooftop area in the target region was determined to be approximately 1,614,520.69 m^2^. This value represents the realistic surface suitable for photovoltaic (PV) system deployment.

Based on the effective area and the obtained solar radiation data, PV power generation was simulated using the professional software PVsyst, employing 300 W_p_ monocrystalline silicon PV modules with dimensions of 1960 mm × 992 mm × 40 mm. The simulation considered module physical characteristics, system layout, shading effects, and regional meteorological conditions to provide accurate estimates of potential electricity output.

As a result, the annual PV electricity generation and the corresponding carbon dioxide (CO_2_) emission reductions for each newly constructed university campus in Chenggong District, Kunming, were quantified. The detailed results are summarized in Table 6, providing a solid quantitative basis for evaluating the potential environmental and energy contributions of distributed rooftop PV systems in the study area.

## 4. Discussion

### 4.1. Methodological Advantages and Comparative Analysis

The proposed multidimensional evaluation framework addresses several critical limitations inherent in traditional rooftop PV assessment methods. First, the integration of a multi-model fusion strategy effectively mitigates the architectural biases of single-model predictions in rooftop segmentation and two-dimensional PV potential estimation. While single-model approaches such as U-Net or Mask R-CNN have shown competence in specific contexts [10,11], they often struggle with generalization across diverse urban rooftop types and complex scenes. Our CESW-TransUNet model, enhanced with convolutional Swin Transformer blocks and multi-scale attention, achieved an IoU of 78.50% on the INRIA dataset, outperforming TransUNet by 2.27 percentage points. This demonstrates the model’s improved capability in capturing both fine-grained details and global contextual features.

Second, the proposed residual fusion strategy represents a significant improvement over conventional majority voting methods [12,13]. By performing reliability screening at the patch level and incorporating only high-confidence auxiliary predictions, this approach reduces error propagation and noise incorporation. The residual fusion strategy elevated the IoU to 79.85% with a 78% reduction in standard deviation compared to full-range fusion, underscoring its effectiveness in handling spatial heterogeneity and improving decision stability.

Third, the incorporation of physical constraints—such as dynamic inter-building shading, rooftop equipment occupancy, and installation suitability—enables a transition from simplistic 2D area-based estimates to a more realistic 3D-aware assessment. Traditional methods often overlook these factors, leading to systematic overestimation of PV potential [33]. By integrating Ecotect-based shadow analysis and statistical correction for equipment coverage, our framework provides a more accurate and practically applicable evaluation.

### 4.2. Significance of the Findings

The empirical results from the case study in Chenggong District, Kunming, demonstrate the practical relevance and robustness of the proposed framework. After incorporating all physical constraints, the effective rooftop area was calculated to be approximately 1.61 km^2^, significantly lower than the 2.21 km^2^ estimate derived from traditional methods that neglect physical constraints such as dynamic shading, rooftop equipment occupancy, and installation suitability [14,15]. Using the unadjusted rooftop area from these traditional methods, PVsyst simulations predicted an annual grid-connected electricity potential of approximately 549,406 MWh, which represents a clear overestimation of the actual generation potential.

In contrast, the simulation based on the effective area adjusted by our framework—considering these physical constraints—resulted in a more realistic annual generation of 430,232 MWh. This indicates that traditional methods, which fail to account for these critical factors, overestimated the annual power generation by approximately 27.7%. This overestimation can be attributed mainly to the inaccurate quantification of shading effects and the exclusion of unsuitable rooftop areas. Our framework effectively captures both diurnal and seasonal shadow coverage variations, aligning with solar geometry principles [19]. These results not only enhance the credibility of rooftop PV planning but also provide a solid quantitative foundation for urban-scale carbon reduction strategies.

### 4.3. Strengths and Limitations

The study presents an integrated workflow that combines advanced deep learning with physical modeling to provide a scalable and transferable solution for urban renewable energy planning. The proposed CESW-TransUNet model maintains high accuracy while reducing computational cost by 26.61% compared to TransUNet. The residual fusion strategy further enhances the precision of building outline extraction without significantly increasing inference time, demonstrating strong practical potential.

However, several limitations should be acknowledged. First, the shadow simulation relied on building height data derived from POI databases rather than high-resolution LiDAR measurements, which may introduce errors in geometric accuracy. Future integration of UAV or LiDAR data could improve modeling precision [34]. Second, the multi-model fusion strategy, although effective, requires training and maintaining multiple networks, leading to high computational costs and limiting real-time applicability. Finally, the rooftop equipment ratio was estimated via statistical sampling, which, though practical, may not capture full spatial variability. A dedicated deep learning-based equipment detection model could offer higher accuracy in future work. Additionally, temporal meteorological variations—such as cloud cover and seasonal irradiance fluctuations—were not fully dynamically incorporated into the radiation model. Future iterations could benefit from real-time weather data integration to enhance temporal resolution and prediction stability.

## 5. Conclusions

This study successfully developed a multidimensional evaluation framework that significantly enhances the accuracy of urban rooftop photovoltaic potential assessment by integrating an improved deep learning segmentation model (CESW-TransUNet), a novel residual-fusion strategy, and physics-based constraint simulations. The primary finding is that the proposed framework effectively mitigates the systematic overestimation prevalent in conventional 2D area-based methods, reducing the overestimation of annual energy generation by 27.7% in our case study. The CESW-TransUNet model achieved a rooftop segmentation IoU of 78.50% on the INRIA dataset, a 2.27 percentage point improvement over the TransUNet baseline. The subsequent residual fusion of multiple models further elevated the segmentation accuracy, reaching an IoU of 79.85% and demonstrating superior robustness against spatial heterogeneity.

The practical contribution of this work lies in its provision of a reliable, end-to-end tool for urban planning and energy policy. By coupling the high-precision segmentation results with dynamic shading analysis and equipment occupancy corrections, we derived a realistic effective rooftop area of 1.61 km^2^ for the study area in Chenggong District, Kunming. This area translates to a potential annual electricity generation of 430,232 MWh and a carbon emission reduction of 270,992 tCO_2_, offering quantifiable and actionable data for achieving dual-carbon goals.

The current study relies on building height data derived from Points of Interest (POI) rather than high-resolution Light Detection and Ranging (LiDAR) data, which introduces certain uncertainties in shadow simulation. Furthermore, the multi-model fusion strategy remains computationally expensive, and the statistical sampling approach for estimating rooftop equipment occupancy leaves room for further refinement. Future research will focus on several key enhancements: integrating LiDAR and unmanned aerial vehicle (UAV) data to improve the accuracy of rooftop geometric feature extraction; developing deep learning-based models for automated equipment detection and precise area estimation, thereby replacing the current statistical sampling method; and optimizing the multi-model fusion pipeline to reduce computational overhead. These advancements are expected to significantly enhance the accuracy, efficiency, and applicability of the framework across diverse urban environments, thereby providing robust support for the precise and scalable deployment of renewable energy systems.

## Figures and Tables

**Table 1:** Formulas for evaluation metrics.

Metric	Formula
Accuracy	$P_{a}=\frac{T_{P}+T_{N}}{T_{P}+T_{N}+F_{P}+F_{N}}$
Intersection over Union (IoU)	$IoU=\frac{T_{P}}{T_{P}+F_{P}+F_{N}}$
Precision	$P_{r}=\frac{T_{P}}{T_{P}+F_{P}}$
Recall	$R_{e}=\frac{T_{P}}{T_{P}+F_{N}}$
F1 Score/F1	$F1=\frac{2\times \left(R_{e}\times P_{r}\right)}{R_{e}+P_{r}}$

**Table 2:** Hyperparameter settings.

Parameter	Value/Description
Batch size	4
Number of iterations	40,000
Learning rate (lr)	0.01/dynamically adjusted
Optimizer	SGD
Momentum	0.9
Activation function	ReLU, GELU
Weight decay	1 × 10^−5^

**Table 3:** Performance comparison of different semantic segmentation networks on rooftop segmentation.

Model	Pa (%)	IoU (%)	Pr (%)	Re (%)	F1 (%)	Parameters (M)	FLOPS (G)	FPS
U-Net [24]	93.71	72.12	85.40	82.27	83.80	15.17	130.71	30.86
DeepLabv3+ [25]	94.38	75.48	86.28	83.88	85.18	42.40	46.81	31.06
PSPNet [26]	94.42	75.73	84.28	86.40	85.34	57.10	53.59	40.16
FCN [27]	93.69	70.91	88.88	77.81	82.98	28.15	27.64	88.50
UPerNet [28]	94.51	75.17	87.75	83.98	85.82	40.75	115.43	27.93
MobileNet [29]	93.38	71.63	82.45	84.52	83.47	11.60	61.85	69.93
DNLNet [30]	93.83	72.31	83.93	86.52	85.21	14.27	79.14	18.55
APCNet [31]	94.01	72.27	89.49	78.97	83.90	218.84	69.97	30.67
SegFormer [32]	94.50	74.89	88.49	82.98	85.64	32.13	51.78	93.46
TransUNet [16]	95.33	76.23	87.92	84.84	86.35	123.84	85.67	26.18
Swin-T [17]	95.38	77.92	88.53	88.14	88.33	33.22	65.15	23.04
Ours	95.47	78.50	88.60	88.12	88.36	90.89	67.69	32.69

**Table 4:** Ablation study of the proposed modules on the INRIA dataset for rooftop segmentation.

Configuration	SW-Block	CESW-Block	MSCA	GCFF	Pa (%)	IoU (%)	Pr (%)	Re (%)	F1 (%)
Baseline	×	×	×	×	93.85	76.32	86.40	86.75	86.07
+ SW-Block	√	×	×	×	94.38	77.09	87.14	87.01	87.08
+ CESW-Block	×	√	×	×	95.20	78.25	88.35	87.90	87.83
+ MSCA	×	×	√	×	94.35	77.18	87.05	87.30	87.12
+ GCFF	×	×	×	√	94.62	77.41	87.51	87.29	87.40
Full Model	√	√	√	√	95.47	78.50	88.60	88.12	88.36

√: Module included; ×: Module excluded.

**Table 5:** Comparative rooftop segmentation performance of different multi-model fusion methods.

Method	Pa (%)	IoU (%)	Pr (%)	Re (%)	F1 (%)
TransUNet [16]	95.33	76.23	87.92	84.84	86.35
Swin-T [17]	95.38	77.92	88.53	88.14	88.33
CESW-TransUNet	95.47	78.50	88.60	88.12	88.36
Full-range majority voting fusion [12,13]	95.36 ± 0.15	77.56 ± 0.82	88.37 ± 0.40	87.29 ± 0.65	87.83 ± 0.47
Residual fusion	95.81 ± 0.09	79.85 ± 0.18	89.42 ± 0.15	89.15 ± 0.20	89.28 ± 0.12

**Table 6:** Annual Photovoltaic Power Generation Analysis for Each Campus.

University	Effective Rooftop Area (km^2^)	Grid-Connected Electricity (MWh)	Carbon Emission Reduction (tCO_2_)
Yunnan University (YNU)	0.2995	79,596	50,136
Kunming University of Science and Technology (KUST)	0.2426	64,541	40,652
Yunnan Normal University (YNU)	0.2894	77,414	48,761
Yunnan Minzu University (YMU)	0.2993	79,635	50,160
Yunnan Jiaotong University (YJTU)	0.0732	19,559	12,320
Yunnan Open University (YOU)	0.0864	23,081	14,538
Kunming Medical University (KMU)	0.1466	38,963	24,542
Yunnan University of Chinese Medicine (YUNCM)	0.0947	25,302	15,937
Yunnan Arts University (YAU)	0.0828	22,141	13,946

## Data Availability

The raw data supporting the conclusions of this article will be made available by the authors on request.
